# Mc Donalds’ model of deliverance of opioid substitution treatment in COVID-19 crisis in opioid belt in rural India : A retrospective - comparative study

**DOI:** 10.1192/j.eurpsy.2021.1531

**Published:** 2021-08-13

**Authors:** G. Uppal

**Affiliations:** Psychiatry, Sankalp Drug Dependence Center, Tarn Taran, Tarn Taran, India

**Keywords:** COVID-19, Buprenorphine -naloxone, Substance, opioid substitution therapy

## Abstract

**Introduction:**

COVID-19 has been declared as a pandemic by the World Health Organization on March, 2020. Opioid substitution therapy (OST) for opioid-dependent patients is an evidence-based, effective outpatient (OPD) based treatment maintained on medications(, buprenorphine-naloxone combination). These are difficult times for patients with addictive disorders. Authors came up with an idea of implementing “walk through” out patient model inspired by “Drive through” model of Mc Donalds in which Doctor and Nurses run open space OPD near to Medical record section to reduce the waiting time of patient, quick delivery and minimize droplet exposure.

**Objectives:**

To study the efficacy of Mc Donald’s model of OST in OST centre in Tarn Taran, india.

**Methods:**

This was a restrospective-comparative study. We studied records of our patients using streatment as usual from September 2019 to March 2020 and using Mc Donald’s model (From April 2020 to October, 2020) for a period of 6 months during and before lockdown.

**Results:**

The average number of patients attending OST clinics during Covid lockdown was 352.8. The compliance rates significantly improved (57.82%), The drop out rates were much lower (20.78%), using Mc Donald’s model than usual treatment.

**Conclusions:**

As per our knowledge, this study is first of its kind to study the efficacy of OST drug deliverance in pre Covid and Covid times and suggest, the new findings which can be inculcated in the other OSTs.

